# Correlation Between Patatin-Like Phospholipase Domain-Containing Protein 3 Gene Polymorphisms and Liver Cirrhosis in a Chinese Han Population With Chronic Hepatitis B

**DOI:** 10.5812/hepatmon.18943

**Published:** 2014-08-17

**Authors:** Jin Tong, Jinjun Guo, Jun Hu, Sihui Hou, Yu Zhang, Qingling Li

**Affiliations:** 1 Institute of Life Sciences, Chongqing Medical University, Chongqing, China; 2 Department of Gastroenterology and Hepatology, The Second Affiliated Hospital of Chongqing Medical University, Chongqing, China

**Keywords:** Hepatitis B, Chronic, Liver Cirrhosis, Single Nucleotide Polymorphisms

## Abstract

**Background:**

A single nucleotide polymorphism (SNP) of patatin-like phospholipase domain-containing 3 (*PNPLA3*) genes (rs738409) is associated with the severity of fibrosis and cirrhosis in patients with fatty liver disease. However, in a small group of Italian patients, there was no significant correlation between the rs738409 SNP and hepatitis B virus (HBV) infection-associated liver cirrhosis.

**Objectives:**

This study aimed to investigate whether PNPLA3 polymorphisms are a risk factor for liver cirrhosis in a Chinese Han population with chronic hepatitis B (CHB).

**Patients and Methods:**

The study population consisted of 344 Chinese Han patients with CHB, among which 203 presented with liver cirrhosis (LC group) and 141 had no sign of liver cirrhosis (CHB group). TaqMan genotyping assay was used to investigate the association of two PNPLA3 SNPs (rs738409 and rs2281135) with the risk of liver cirrhosis.

**Results:**

The allele and genotype distributions of PNPLA3 rs738409 and rs2281135 were not significantly different between the CHB and LC groups. After segregation on the basis of sex, no significant correlation between PNPLA3 (rs738409 and rs2281135) genotypes/alleles and liver cirrhosis was detected. Moreover, none of the haplotypes in PNPLA3 (rs738409 and rs2281135) was found to be statistically different between the two groups.

**Conclusions:**

Our results showed no association between PNPLA3 polymorphisms (rs738409 and rs2281135) and the susceptibility to HBV-related liver cirrhosis in a Chinese Han population.

## 1. Background

About 400 million people are infected with hepatitis B virus (HBV) worldwide and almost one-third of them are Chinese (1, 2). Cirrhosis is one of the most frequent complications of chronic HBV infection (CHB) (3). Progressive hepatic fibrosis and cirrhosis develop in 20% to 30% of patients with CHB (3, 4). There is compelling evidence that host genetic factors are involved in the progression of HBV-related liver fibrosis (5-7). Several single nucleotide polymorphisms (SNPs) within host genes and/or gene regions such as *IL-10, TANK, *and* TGFB1* have been found to be closely associated with progressive HBV-induced liver fibrosis (5-7). However, these previous studies have shown various biases and some yield contradictory results.

Patatin-like phospholipase domain-containing 3 (*PNPLA3*) genes encodes a 481-amino acid triacylglycerol lipase conserved from potatoes to humans (8). It is highly expressed in the liver, with ten-fold higher expression in comparison to adipose tissue (8). Recent studies have reported that a non-synonymous sequence variation (rs738409 C > G), which encodes an isoleucine to methionine substitution at position 148 (I148M) in PNPLA3, is associated with the susceptibility to fatty liver disease (FLD) and FLD-related liver fibrosis (9-12). This polymorphism is also related to liver steatosis and fibrosis in patients with chronic hepatitis C (13).

Liver steatosis is a common pathologic condition in CHB, with the prevalence varying from 27% to 57% (14-16). Metabolic syndrome, which is characterized by a cluster of metabolic abnormalities, is an important risk factor of non-alcoholic steatohepatitis and non-alcoholic FLD (NAFLD) (11). A recent large-scale study has revealed that metabolic syndrome increases the risk of severe fibrosis and cirrhosis in patients with CHB (17). Given the associations of PNPLA3 polymorphisms with liver steatosis and fibrosis in patients with chronic hepatitis C, we proposed that PNPLA3 polymorphisms might contribute to the progression of liver fibrosis and cirrhosis in CHB (13). 

Although here was no correlation between the rs738409 polymorphism and HBV-related liver cirrhosis (18), due to a small sample size (76 patients with HBV-related cirrhosis versus 428 control subjects), it lacked sufficient power to determine the effect of PNPLA3 polymorphisms on HBV-related cirrhosis. The association between PNPLA3 polymorphism rs2281135 and alanine aminotransferase (ALT) levels was recently reported in Mexican-American population (19). As shown in Table 1, two PNPLA3 SNPs, namely, rs738409 and rs2281135, are in tight linkage disequilibrium in East-Asian population. However, their roles in the progression of HBV-related liver cirrhosis are largely unknown.

**Table 1.** Linkage Disequilibrium of the Two PNPLA3 Single Nucleotide Polymorphisms ^a^

Population	rs738409 (Minor alleles, Frequency)	rs2281135 (Minor alleles, Frequency)	LD of rs738409-rs2281135 (D’, r2)
**CHB + JPT**	G, 0.223	A, 0.388	0.976, 0.953

^a^ JPT, Japanese in Tokyo, Japan; and CHB, Han Chinese in Beijing, China. These reference populations were genotyped in the HapMap study (http://hapmap.ncbi.nlm.nih.gov/)

## 2. Objectives

The present study aimed to determine whether PNPLA3 polymorphisms (rs738409 and rs2281135) have an adverse influence on HBV-related liver cirrhosis in a Chinese Han population.

## 3. Patients and Methods

### 3.1. Patients

A total of 344 Chinese Han patients with CHB were enrolled in this study. These patients had a permanent residence in Chongqing, China and had no hereditary relationship. CHB was defined as the presence of hepatitis B surface antigen (HBsAg) and elevated serum ALT and HBV DNA for at least six months (20). The patients were divided into two groups according to the presence or absence of liver cirrhosis (LC group; number = 141; and CHB group; number = 203, respectively). The diagnosis of liver cirrhosis in patients with CHB was made based on clinical signs (e.g. ascites and esophageal varices) and imaging findings on ultrasonography, computed tomography, and magnetic resonance imaging. We excluded the patients with any other pathology, such as hepatitis C virus (HCV) or human immunodeficiency virus (HIV) infection, drug-induced hepatitis, autoimmune liver disease, non-HBV liver cirrhosis, and hepatocellular carcinoma (HCC). 

Written informed consent was obtained from each subject and the Ethics Committee of the Second Affiliated Hospital of Chongqing Medical University (Chongqing, China) approved the study protocol.

### 3.2. Physical and Biochemical Evaluation

A questionnaire was used to record patient information including age, sex, alcohol consumption, and history of diabetes. Serum total bilirubin (T-Bil), gamma-glutamyl transferase (GGT), albumin level (ALB), HBV markers, aspartate aminotransferase (AST), ALT, and HBV-DNA levels were measured by standard clinical laboratory techniques.

### 3.3. DNA Extraction and Genotyping

High-molecular-weight genomic DNA was isolated from peripheral whole blood leukocytes using a Wizard Genomic DNA Purification Kit according to the manufacturer's protocol (Promega, Madison, WI, USA). Two predesigned TaqMan probes (Applied Biosystems, Foster City, CA, USA) were used for genotyping of rs738409 (C_7241_10) and rs2281135 (C_15875080_10). Genotyping was performed on a CFX96 Real-Time PCR system (Bio-Rad, Pleasanton, CA, USA).

### 3.4. Statistical Analysis

Values with a normal distribution were expressed as mean ± standard deviation, while variables that were not distributed normally were shown as median (range). Continuous variables were compared using the Mann-Whitney U test. Chi squared test was used to compare the differences in categorical variables (i.e. sex, alcohol consumption, and diabetes status). Chi squared test was also used to assess whether the genotypes were in Hardy-Weinberg equilibrium (HWE) and to test the associations between different genotypes or alleles and the risk of HBV-related liver cirrhosis. Binary logistic regression was conducted to estimate the relative risk of SNPs and adjusted odds ratios (ORs) and their 95% confidence intervals (95% CI) were calculated. P value < 0.05 was considered statistically significant. Haplotype analysis was performed using the Haploview 4.2 software (http://www.broad.mit.edu/haploview/haploview) and other statistical analyses were done using the SPSS version 19.0 (SPSS Inc., Chicago, IL, USA).

## 4. Results

### 4.1. General Characteristics

Demographic and laboratory parameters of all the subjects are shown in Table 2. The distribution of sex, drinking behavior, or history of diabetes was not statistically different between the two groups (P > 0.05). The mean age of patients without liver cirrhosis was significantly lower than that of those with liver cirrhosis (P < 0.001). In addition, the two groups differed significantly in laboratory results including serum ALT, AST, GGT, ALB, and HBV DNA levels (P < 0.001).

**Table 2.** Demographic and Clinical Characteristics of the Studied Population ^a^

Parameters	CHB (n = 141)	LC (n = 203)	P Value
**Male/Female**	96/45	144/59	0.571 ^b^
**Age, y**	43.65 ± 14.31	50.05 ± 11.56	< 0.001 ^c^
**Diabetes, yes/no**	10/131	25/178	0.115 ^b^
**Alcohol > 30 g/d, yes/no**	18/123	30/173	0.596 ^b^
**ALT, IU/L**	164.5 (6-1940)	41 (9-1797)	< 0.001 ^c^
**AST, IU/L**	106.5 (14-1657)	56 (16-1638)	< 0.001^c^
**GGT, IU/L**	97 (6-1957)	37 (1.4-391)	< 0.001^c^
**T-Bil, μmol/L**	27 (0-853)	30.9 (2.2-653)	0.252 ^c^
**ALB, g/L**	36.6 (0-527.7)	31 (0-305.9)	< 0.001 ^c^
**HBV-DNA, copies/ml**	9.76E4 (0-2.73E8)	1.67E3 (0-1.16E8)	< 0.001 ^c^

^a^ Abbreviations: ALT, serum alanine aminotransferase; AST, aspartate aminotransferase; GGT, gamma-glutamyl transferase; T-Bil, serum total bilirubin; ALB, serum albumin; CHB, chronic hepatitis B group; and LC, liver cirrhosis group. Continuous variables are reported as mean ± SD or medians (range) and categorical variables as frequencies.

^b^ Obtained P value by the Pearson's chi-square test.

^c^ Obtained P value by the Mann-Whitney U test.

**Table 3.** Association Analysis Between Liver Cirrhosis and Chronic Hepatitis B ^a^

Polymorphisms	Alleles	CHB (n = 141)	LC (n = 203)	LC vs. CHB	
				P value	P value/OR (95%CI) ^b,c^
**rs738409**	CC	62 (0.44)	79 (0.39)	Ref	
	CG	55 (0.39)	91 (0.45)	0.278	0.119/1.532 (0.897-2.617)
	GG	24 (0.17)	33 (0.16)	0.81	0.831/0.927 (0.46-1.868)
	GG + GC^d^	79 (0.56)	124 (0.61)	0.348	0.258/1.326 (0.813-2.164)
	C	179 (0.63)	249 (0.61)	Ref	
	G	103 (0.37)	157 (0.39)	0.568	0.718/1.067 (0.75-1.518)
**rs2281135**	GG	61 (0.43)	74 (0.36)	Ref	
	AG	55 (0.39)	94 (0.46)	0.157	0.066/1.458 (0.968-2.839)
	AA	25 (0.17)	35 (0.17)	0.648	0.895/1.048 (0.524-2.093)
	AA + AG ^d^	80 (0.57)	129 (0.64)	0.203	0.139/1.450 (0.886-2.374)
	G	177 (0.62)	242 (0.60)	Ref	
	A	105 (0.37)	164 (0.40)	0.403	0.483/1.134 (0.798-1.61)

^a^ Data were presented as No. (%).

^b^ P value or OR after adjustment. The statistical analysis was performed using the Pearson's Chi square test, and using binary logistic analysis to adjust age, ALT, AST, HBV DNA, ALB, and GGT.

^c^ Abbreviations: CI, confidence interval; CHB, chronic hepatitis B group; LC, liver cirrhosis group; and OR, odds ratio.

^d^ At-risk genotype.

**Table 4.** Association Analysis Between Liver Cirrhosis and Chronic Hepatitis B After Segregation on the Basis of Sex ^a^

	Alleles	CHB, n = 45	LC, n = 59	LC vs. CHB	
				P value	P value /OR (95%CI) ^b^
**Female**					
**rs738409**	CC	19 (0.42)	23 (0.39)	Ref	
	CG	18 (0.4)	25 (0.42)	0.754	0.606/0.739 (0.235-2.33)
	GG	8 (0.18)	11 (0.19)	0.82	0.671/0.727 (0.166-3.179)
	GG + GC ^c^	26 (0.58)	36 (0.61)	0.739	0.577/0.763 (0.25-2.164)
	C	56 (0.62)	57 (0.55)	Ref	
	G	34 (0.38)	47 (0.45)	0.296	0.489/0.778 (0.381-1.586)
**rs2281135**	GG	19 (0.42)	20 (0.34)	Ref	
	AG	18 (0.4)	26 (0.44)	0.475	0.709/0.801 (0.251-2.560)
	AA	8 (0.18)	13 (0.22)	0.43	0.982/0.983 (0.229-4.215)
	AA + AG ^c^	26 (0.58)	39 (0.66)	0.385	0.773/0.852 (0.287-2.53)
	G	56 (0.62)	66 (0.56)	Ref	
	A	34 (0.38)	52 (0.44)	0.361	0.798/0.912 (0.449-1.852)
**Male**					
	**Alleles**	**CHB, n = 96**	**LC, n = 144**	**LC vs. CHB**	
				**P value**	**P value /OR (95%CI) ** ^**b,c**^
**rs738409**	CC	43 (0.45)	56 (0.39)	Ref	
	CG	36 (0.38)	66 (0.46)	0.237	0.102/1.713 (0.899-3.263)
	GG	17 (0.18)	22 (0.15)	0.987	0.949/0.922 (0.398-2.132)
	GG + GC ^d^	53 (0.55)	88 (0.61)	0.363	0.229/1.433 (0.798-2.575)
	C	122 (0.64)	178 (0.62)	Ref	
	G	70 (0.36)	110 (0.38)	0.7	0.493/1.150 (0.771-1.714)
**rs2281135**	GG	43 (0.45)	54 (0.38)	Ref	
	AG	36 (0.38)	68 (0.47)	0.159	0.124/1.656 (0.87-3.15)
	AA	17 (0.18)	22 (0.15)	0.937	0.833/0.914 (0.394-2.121)
	AA + AG ^d^	53 (0.55)	90 (0.63)	0.259	0.261/1.401 (0.778-2.521)
	G	122 (0.64)	176 (0.61)	Ref	
	A	70 (0.36)	112 (0.39)	0.591	0.529/1.137 (0.763-1.693)

^a^ Data were presented as No. (%).

^b^ P value or OR after adjustment. The statistical analysis was performed using Pearson's Chi square test, and using binary logistic analysis to adjust age, ALT, AST, HBV DNA, ALB, and GGT.

^c^ Abbreviations: CI, confidence interval; CHB, chronic hepatitis B group; LC, liver cirrhosis group; and OR, odds ratio.

^d^ At-risk genotype.

### 4.2. Association of PNPLA3 Haplotypes With Liver Cirrhosis

Based on the Asian HapMap data (http://hapmap.ncbi.nlm.nih.gov/), both rs738409 and rs2281135 are in tight linkage disequilibrium. Haplotype analysis was achieved using the Haploview 4.2 software. As shown in Table 5, none of the three studied haplotypes (C-G, G-A, and C-A) was significantly associated with HBV-related liver cirrhosis.

**Table 5.** Association of PNPLA3 Haplotypes With Liver Cirrhosis ^a^

PNPLA3 Haplotypes	LC vs. CHB		
	rs2281135	Frequency	P value
**rs738409**			
**C**	G	0.606	0.3348
**G**	A	0.375	0.6598
**C**	A	0.016	0.1214

^a^ Abbreviation: CHB, chronic hepatitis B group; and LC, liver cirrhosis group.

## 5. Discussion

PNPLA3 belongs to the patatin-like phospholipase family. It is frequently upregulated in response to feeding and during adipocyte differentiation and downregulated under fasting conditions (21). The biological function of PNPLA3 in humans has not been very clear yet; however, in vitro studies with recombinant human PNPLA3 have shown that it is involved in the transacylation of acylglycerols via the acyl-CoA pathway and the hydrolysis of triglycerides (22). Previous studies have demonstrated the association of PNPLA3 rs738409 C > G (I148M) with the development and progression of FLD (9-12). The GG genotype was shown to be associated with an increased risk of inflammation and cirrhosis. In patients with chronic hepatitis C, the PNPLA3 rs738409 polymorphism was strongly associated with advanced hepatic fibrosis (13). In China, the most common cause of liver cirrhosis is CHB (2). Therefore, in this study we focused on the association between PNPLA3 polymorphisms and the risk of HBV-related hepatic cirrhosis.

We found that PNPLA3 SNPs and haplotypes (rs7384095 and rs228113) were not significantly associated with the risk of liver cirrhosis in patients with CHB. To the best of our knowledge, this is the first report investigating the association of PNPLA3 polymorphisms with HBV-related liver cirrhosis in a Chinese Han population. These results were consistent with a recent study that has reported no significant correlation between rs738409 and the susceptibility to HBV-related liver cirrhosis in a small group of Italian patients (18). Similarly, there is no association between PNPLA3 rs738409 genotype and hepatic cirrhosis in Japanese patients infected with HCV (23). However, rs738409 in PNPLA3 is strongly associated with alcoholic cirrhosis in Mestizo subjects (10). These studies suggest that PNPLA3 polymorphisms may have a major influence on liver diseases due to metabolic or alcoholic factors rather than viral infection. No significant correlation is found between the presence of steatosis and progression of fibrosis in CHB (20-26). These findings suggest that lipid metabolism may be rarely involved in the pathogenesis of HBV-related cirrhosis. However, there is evidence that liver steatosis is independently associated with the severity of fibrosis in patients with CHB (27). The contrasting obtained results may be explained by differences in the geographic origin and demographic characteristics of study population. Our results, together with the study by Falleti et al. confirmed that there was no strong association between liver steatosis and cirrhosis in patients with CHB (18).

Our data revealed a significant association between age and liver cirrhosis in patients with HBV infection. This result was consistent with a previous study where older age has been identified as an independent predictor of cirrhosis development in Italian patients with CHB (28). However, after adjusting for age, no significant correlation between PNPLA3 genotypes/alleles and liver cirrhosis was detected in our patients. These results confirmed the little effect of PNPLA3 polymorphisms on HBV-related liver cirrhosis. Clinical experience shows that men are more prone to HBV-related cirrhosis than women are. Moreover, earlier studies have revealed that the PNPLA3 SNP (rs738409) has a stronger associations with elevated ALT and AST levels in males than in females (19). The PNPLA3 transcription has been found to be regulated by sterol regulatory element binding protein 1c (SREBP-1c) (29). SREBP-1c is a direct target gene of the liver X receptor (LXR)/retinoid X receptor (RXR) heterodimer (25). Transcriptional regulation of PNPLA3 requires both SREBP-1c and LXR/RXR (21). Estrogen can interfere with the expression of both LXR and SREBP-1c (22). Therefore, estrogen might influence the transcription of PNPLA3 via downregulation of LXR and SREBP-1c. However, our data revealed that after stratification of all subjects based on sex, PNPLA3 polymorphisms had no significant association with HBV-related cirrhosis. The unexpected results may be due to the relatively small sample size and selection bias. Further studies involving a larger cohort of patients from multiple centers are needed to address this issue. 

In conclusion, our results suggest that the PNPLA3 polymorphisms (rs738409 and rs2281135) and haplotypes are not associated with the susceptibility to HBV-related liver cirrhosis in a Chinese Han population. However, this study cannot completely rule out the possible association of PNPLA3 SNPs with to HBV infection-related liver cirrhosis. Further studies with a larger sample size would provide more definitive information. The sex-specific association of PNPLA3 SNPs with HBV-related cirrhosis also deserves further investigation.
